# “Osteomicrobiology”: The Nexus Between Bone and Bugs

**DOI:** 10.3389/fmicb.2021.812466

**Published:** 2022-01-25

**Authors:** Asha Bhardwaj, Leena Sapra, Abhay Tiwari, Pradyumna K. Mishra, Satyawati Sharma, Rupesh K. Srivastava

**Affiliations:** ^1^Department of Biotechnology, All India Institute of Medical Sciences (AIIMS), New Delhi, India; ^2^Centre for Rural Development & Technology, Indian Institute of Technology (IIT), New Delhi, India; ^3^Department of Molecular Biology, ICMR-National Institute for Research in Environmental Health, Bhopal, India

**Keywords:** osteomicrobiology, bone health, gut microbiota, probiotics, prebiotics

## Abstract

A growing body of scientific evidence supports the notion that gut microbiota plays a key role in the regulation of various physiological and pathological processes related to human health. Recent findings have now established that gut microbiota also contributes to the regulation of bone homeostasis. Studies on animal models have unraveled various underlying mechanisms responsible for gut microbiota-mediated bone regulation. Normal gut microbiota is thus required for the maintenance of bone homeostasis. However, dysbiosis of gut microbiota communities is reported to be associated with several bone-related ailments such as osteoporosis, rheumatoid arthritis, osteoarthritis, and periodontitis. Dietary interventions in the form of probiotics, prebiotics, synbiotics, and postbiotics have been reported in restoring the dysbiotic gut microbiota composition and thus could provide various health benefits to the host including bone health. These dietary interventions prevent bone loss through several mechanisms and thus could act as potential therapies for the treatment of bone pathologies. In the present review, we summarize the current knowledge of how gut microbiota and its derived microbial compounds are associated with bone metabolism and their roles in ameliorating bone health. In addition to this, we also highlight the role of various dietary supplements like probiotics, prebiotics, synbiotics, and postbiotics as promising microbiota targeted interventions with the clinical application for leveraging treatment modalities in various inflammatory bone pathologies.

## Introduction

**FIGURE 1:**
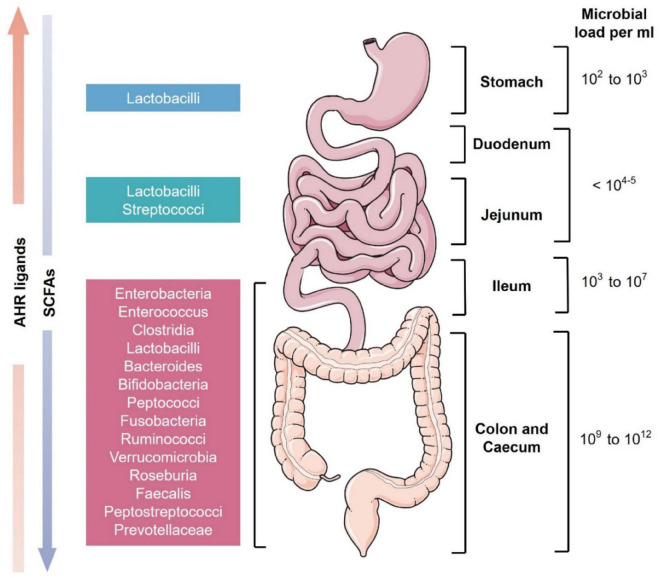
Schematic representation of microbial diversity throughout the human gastrointestinal tract. The stomach has the least microbial diversity whereas the colon and cecum are the most diverse (Ahmed et al., 2007). These microbes produce various essential secondary metabolites from the diet like short-chain fatty acids (SCFAs) and aryl hydrocarbon receptor (AhR) ligands. SCFAs are present in higher levels in the colon while AhR ligands are more concentrated in the small intestine (Mowat and Agace, 2014).

Microbiota is the collection of microorganisms such as bacteria, viruses, archaea, fungi, protozoa, and eukaryotes that exist inside or outside the host. Although microbes are present on the entire host mucosal surface a majority of them reside in the gastrointestinal tract and are referred to as gut microbiota (GM) (Sommer and Bäckhed, 2013). The human GM consist of over 100 trillion microbes that encode more than 3.3 million genes (Qin et al., 2010; Rinninella et al., 2019). These microbes have coevolved with humans and provide numerous health benefits *via* the regulation of various biological processes (Eckburg, 2005). The human gut is primarily dominated by the *Firmicutes* and *Bacteroidetes* phyla which constitute over 90% of the intestinal microbiota (Eckburg, 2005; Figure 1). Other microbial phyla that are present in minor proportion are *Proteobacteria*, *Actinobacteria*, *Fusobacteria*, and *Verrucomicrobia* (Eckburg, 2005). Development of GM takes place after birth. At birth, neonates are devoid of any microbiota and acquire it only after birth when exposed to a vast array of microbes. The microbial composition of the infants depends on the mode of delivery (Dominguez-Bello et al., 2010). Infants born by vaginal delivery have a microbial composition similar to the mother’s vagina consisting of mainly *Lactobacillus*, *Prevotella*, or *Sneathia* spp. However, babies born by C-section are depleted of bacterial species from the mother’s vagina (Dominguez-Bello et al., 2010). They acquire most of the microbiota from the mother’s skin and from the hospital which is dominated by *Staphylococcus*, *Corynebacterium*, and *Propionibacterium* spp. (Dominguez-Bello et al., 2010). Microbial composition is also affected by genetic and environmental factors (Schroeder and Bäckhed, 2016). Breastfeeding and antibiotic treatment also affect the microbiota composition during infancy. Later in life, the microbial composition is shaped by the type, quality, and quantity of diet. GM promotes human health and plays a very significant role in the regulation of immune and metabolic homeostasis (Thursby and Juge, 2017). Research from the past decade has revealed the effect of GM in the regulation of various physiological functions related to human health and its association with several diseases. GM enhances the extraction of energy from food, increases nutrient absorption, induces immune system development, and prevents the invasion of pathogens through the intestinal epithelial cells (IECs). GM produces various secondary metabolites such as short-chain fatty acids (SCFAs) and aryl hydrocarbon receptor (AhR) ligands which have a role in the regulation of several physiological functions related to human health. However, modifications in GM composition result in the progression of various gastrointestinal and metabolic disorders such as irritable bowel syndrome (IBS), inflammatory bowel disease (IBD), colorectal cancer, obesity, and type 2 diabetes (Guinane and Cotter, 2013; Wang et al., 2017). The role of GM in regulating human health is now emerging as one of the latest trends in integrative biology. Interestingly, recent studies have now revealed a nexus between GM and skeletal health. Accumulating evidence from these studies indicates that GM is associated with bone metabolism and a range of metabolic bone disorders. GM is a crucial regulator of bone homeostasis and maintains bone health *via* several mechanisms. Here we review all possible mechanisms through which GM regulates skeletal health starting with a brief discussion about bone.

## Bone

The skeleton is among the largest organs of the human body and constitutes approximately 15% of the total human body weight (Su et al., 2019). Adult human bone is mainly composed of 80% cortical bone and 20% cancellous or trabecular bone (Clarke, 2008). Bone is a highly dynamic and metabolically active connective tissue that continuously undergoes remodeling throughout our life. Bone remodeling occurs with the help of three prime bone cells: bone-forming osteoblasts, bone-resorbing osteoclasts, and the embedded cells in a bone matrix called osteocytes. Osteoblasts are differentiated from mesenchymal stem cells (MSCs) that also give rise to various other types of cells such as adipocytes and chondrocytes (Schett and David, 2010). Differentiation of osteoblasts from MSCs is mainly induced by runt-related transcription factor 2 (Runx2) and its target gene, the Sp7 transcription factor (Srivastava et al., 2018). Osteoblasts produce various extracellular proteins such as type 1 collagen, alkaline phosphatase, osteocalcin, osteopontin, and osteonectin. These proteins deposit between the osteoblasts and the surface of the bone to form a matrix (Dar et al., 2018a).

Osteoclasts are specialized in bone resorption and are also known as bone eaters. Osteoclasts are multinucleated giant cells that are derived from myeloid precursors. Differentiation of osteoclasts depends on two important factors i.e., macrophage colony-stimulating factor (MCSF) and receptor activator of nuclear factor kappa-B ligand (RANKL) (Charles and Aliprantis, 2014). MCSF promotes the proliferation and survival of osteoclast progenitors whereas RANKL stimulates the differentiation of progenitors into osteoclasts (Schett and David, 2010). Osteoclasts attach to the surface of the bone to form a unique structure called a “sealing zone” which creates resorption space that is insulated from the extracellular space. Osteoclasts acidify the resorption space and degrade various organic and mineral contents of bone by releasing the lysosomal enzymes such as cathepsin K and tartrate-resistant acid phosphatase (TRAP) (Walsh et al., 2006). Osteoclasts also modulate their structure to form a ruffled border that increases the surface for transport of active hydrogen ions (H^+^) through a proton pump in the sealing zone (Walsh et al., 2006). Recently, a new subset of bone cells named “Osteomorphs” has been reported. Osteomorphs are formed by the fission of RANKL stimulated multinucleated osteoclasts into daughter cells that *via* fusion can recycle back into osteoclasts. Single-cell RNA sequencing revealed that osteomorphs are different from osteoclasts and express several genes that are associated with bone structure and function (McDonald et al., 2021).

A subset of osteoblasts that are being entrapped in the calcified bone matrix is termed osteocytes. Osteocytes are derived from osteoblasts and are the most abundant and long-lived bone cells that constitute about 95% of the mature bone tissue. Osteocytes indirectly regulate the activity of osteoclasts and osteoblasts *via* the secretion of various regulatory factors. Osteocytes also have a role in calcium and phosphate metabolism. The most important function of osteocytes is that they act as bone mechanosensors (Qin et al., 2020). Osteocytes are believed to send signals to other osteocytes and osteoblasts on the surface of the bone in response to mechanical forces *via* their intricate cellular networks called canaliculi (Walsh et al., 2006).

Apart from these prime cells, there are osteomacs which also have a critical role in regulating bone metabolism. Osteomacs are stellate-shaped cells and account for approximately one-sixth of the total bone marrow cells (Srivastava et al., 2018). Osteomacs are present adjacent to osteoblasts and have a role in bone formation and osteogenic differentiation of MSCs (Chen et al., 2020).

**FIGURE 2:**
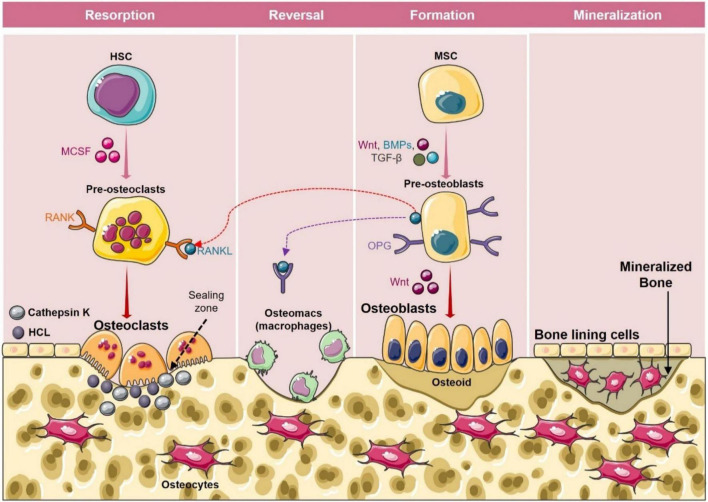
Bone remodeling cycle. Bone remodeling occurs in four phases: (1) Activation phase: In this phase, M-CSF and RANKL promote the differentiation of osteoclast precursors into osteoclasts. (2) Resorption phase: During this phase mature osteoclasts with unique ruffled borders induce resorption of bone by secreting cathepsin K, and H^+^ in the sealing zone. After resorption osteoclasts detach from the surface of the bone and undergo apoptosis. (3) Reversal phase: In the reversal phase osteoblasts’ precursors get differentiated into mature osteoblasts and are recruited to the resorption site. (4) Formation phase: In this phase osteoblasts get occupied in the resorbed lacuna and start depositing the bone matrix. After the formation phase, osteoid gets mineralized and bone surface returns to resting phase with bone lining cells.

Bone remodeling is a dynamic process and requires multiple interactions between osteoblasts and osteoclasts (Figure 2). Osteoblasts positively induce osteoclasts differentiation by secreting RANKL and MCSF and negatively by secreting RANKL decoy receptor osteoprotegerin (OPG) (Schett and David, 2010). Bone remodeling maintains the bone architecture and continuously replaces the old bone with the new one. Bone remodeling maintains bone integrity, restores bone microdamage, and regulates the release of calcium (Ca) and phosphorus (P) during normal host physiology (Dar et al., 2018a). Abnormalities in the bone remodeling process lead to several skeleton deformities. Bone remodeling is a complex process and is regulated by various biochemical and mechanical factors. Major factors that are involved in the regulation of bone remodeling are parathyroid hormone (PTH), estrogen, thyroid hormones, and glucocorticoids. Other factors that are pivotal players in bone remodeling are insulin-like growth factors (IGFs), bone morphogenetic proteins (BMPs), prostaglandins, vitamin D, tumor growth factor (TGF)-β, and cytokines (Hadjidakis and Androulakis, 2006; Dar et al., 2018a). Recent studies particularly on the germ-free (GF) mice have shown that GM is also a critical factor in the regulation of bone remodeling (Yan and Charles, 2017). GM influences the activity of bone cells and alteration in GM composition leads to skeleton manifestations such as osteoporosis, rheumatoid arthritis (RA), and periodontitis (Ibáñez et al., 2019). Recent studies have established the important relationship between GM and bone health and is comprehensively reviewed here.

## Nexus Between Gut Microbiota and Bone Health

GM influences the function of various organs and recently the effect of GM on bone health is catching significant attention. The association between GM and bone was first revealed in the study by Sjogren’s group. They observed that female C57BL/6J GF mice had increased bone mass with decreased number of osteoclasts. GF mice also had a reduced number of CD4^+^ T cells, osteoclast precursors, and inflammatory cytokines than the conventionally raised (CONV-R) mice (Sjögren et al., 2012). The same group in another study using female C57BL/6J GF mice from Pasteur Institute, France observed similar results i.e., female GF mice had increased bone mass than the CONV-R mice as they reported in the previous experiment with C57BL/6J GF mice raised in the gnotobiotic facility at the University of Gothenburg, Sweden (Ohlsson and Sjögren, 2018). Li et al. also observed related results on female C57BL/6J GF mice. They reported that a decrease in bone mass due to inflammatory conditions induced during sex steroid deficiency is mediated by GM. They observed that in GF mice, no bone loss occurred during sex steroid deficiency. In the presence of GM, sex steroid deficiency unregulated the production of osteoclastogenic and inflammatory cytokines such as tumor necrosis factor (TNF)-α, RANKL, and interleukin (IL)-17. However, in GF mice there was no such increase in the level of osteoclastogenic cytokines after sex steroid deficiency (Li et al., 2016). Therefore, GM might enhance bone resorption by increasing the level of inflammatory and osteoclastogenic cytokines as observed by Sjogren’s group. Subsequently, several other studies using mice of the same genetic background i.e., C57BL/6J revealed the different mechanisms involved in GM-mediated bone resorption. Ohlsson et al. (2017) demonstrated that colonization of GM normalized the increased bone mass in GF mice. GM induced the expression of RANKL and TNF-α *via* activation of nucleotide-binding oligomerization domain-containing proteins (NOD)1 and NOD2 signaling which induces osteoclastogenesis and thus bone resorption (Ohlsson et al., 2017). In another study, it was reported that bone marrow-derived mesenchymal stem cells (BMMCs) of GF mice showed high proliferation and osteogenesis. On the other hand, colonization of GF mice with specific pathogen-free (SPF) mice microbiota normalized the proliferation of BMMCs and decreased their osteogenesis (Xiao et al., 2017). Novince et al. (2017) showed that commensal microbiota promotes osteoclastogenesis along with simultaneously suppressing osteoblastogenesis which results in bone loss. Role of GM in inducing bone resorption is also supported by the studies including antibiotics. Low dose administration of antibiotics like penicillin, chlortetracycline and vancomycin at the early age increased the bone mineral density (BMD) of mice (Cho et al., 2012). Another study showed that low dose administration of penicillin enhanced the BMD and bone mineral content (BMC) of female C57BL/6J mice (Cox et al., 2014). However, in humans administration of broad spectrum antibiotics did not show any affect on bone health (Mikkelsen et al., 2018).

Above mentioned studies thus clearly indicate that GM regulates bone mass by promoting bone resorption but in reality, the effect of GM on bone is very complex as various factors are involved in GM-mediated regulation of bone health. These factors could be the strain and gender of mice used in the study. Some studies conducted on male mice of other strains evidenced that GM induces bone formation instead of bone resorption. It is reported that supplementation of *Lactobacillus plantarum* promoted juvenile growth and prevented stunted growth during chronic undernutrition in male BALB/c GF mice by stimulating growth hormone (GH) activity (Schwarzer et al., 2016). In male C57BL/6J mice antibiotic use decreased the BMC which in contrast to that observed in female C57BL/6J mice (Cox et al., 2014). Similarly, Yan et al. reported that long-term colonization of CB6F1 GF mice with microbiota from SPF mice enhances bone mass. Antibiotic treatment on the other hand inhibits bone formation. Yan et al. study showed that the net effect of GM on bone is not only dependent on the strain but partly on the duration of colonization also. They observed that colonization of GF mice for a short duration i.e., for one month severely decreases bone mass whereas for 8 months increases bone formation. Colonization of mice increases the level of IGF-1 in serum which at a shorter duration inhibits bone formation as it promotes osteoclast formation. On the contrary during long-term colonization, it induced bone formation. Also, short-term colonization enhanced the level of osteoclastogenic cytokines such as RANKL, TNF-α, and IL-1β. in both the colon and bone marrow (Yan et al., 2016). These studies thus reflect that GM can have both catabolic as well as anabolic effects depending on the strain, gender, and duration of colonization.

In contrast to the above-mentioned studies, Quach et al. reported that GM reconstitution does not affect the bone health of GF mice. They observed that colonization of GF mice (Swiss Webster and C57BL/6) with human and mice GM did not alter bone mass significantly. Also, there was no change in the number of osteoclasts precursors, T cells and in the expression of inflammatory cytokines after colonization (Quach et al., 2018). One reason that may be responsible for the differences observed in the study of Quach et al. from other studies is the mode of transplantation of microbiota. The method of colonization used by Sjögren et al. (2012) was coprophagy whereas Quach et al. (2018) used the intragastric gavage method. Thus, changes in the methods of colonization could be responsible for varied results. Other factors for the observed differences in results could be the vendors from where the GF mice were purchased and the animal facility where they were kept. Studies have shown that animals from different facilities harbor a different type of GM. Thus, unique microbiota composition at different animal facilities might also determine the effect of colonization on bone health in GF mice.

Various other studies have demonstrated the role of GM composition on bone health. It is observed that undernourished children have perturbed GM composition. Blanton et al. reported that transplantation of microbiota from undernourished children or infants to GF mice induced impaired growth phenotypes in GF mice. On the other hand cohousing of the GF mice that received the microbiota from the undernourished children with that receiving microbiota from healthy children prevented growth impairments in the recipient mice (Blanton et al., 2016). This supports the notion that microbial diversity determines the net effect of GM on bone health. Another study showed that alterations in the GM composition impairs bone mechanical properties and affect bone strength (Guss et al., 2017). Rios-Arce et al. (2020) reported that dysbiosis caused due to antibiotics treatment, induces bone loss in mice. On the other hand, *Lactobacillus reuteri* administration restored GM composition and alleviated bone loss. A recent study highlighted that transplantation of GM from young rats to aged rats having senile osteoporosis alleviated bone loss by restoring the GM composition of aged rats. This study thereby again supports that healthy GM-diversity promotes bone formation (Ma et al., 2021).

Taken together the effect of GM on bone is very complex and conflicting. Nevertheless, these studies point that GM has the potential to regulate bone homeostasis. To dissect and explore the nexus between GM and bone health a new field was proposed i.e., “Osteomicrobiology”, a term coined by Ohlsson’s group (Ohlsson and Sjögren, 2015). In the following sections, we will review various mechanisms involved in the reciprocal regulation between GM and skeletal health.

### Role of Gut Microbiota in Maintaining Intestinal Integrity

The gut is populated by trillions of microorganisms. To prevent the invasion of microbes into intestinal lamina propria, IECs form the barrier between microbes and gut tissues. Barrier dysfunction allows the access of pathogens into the lamina propria resulting in uncontrolled immune response leading to various inflammatory disorders. The gut barrier is maintained by five different types of specialized epithelial cells. These cells are goblet cells, Paneth cells, microfold (M) cells, enteroendocrine cells, and absorptive enterocytes. Goblet cells secrete mucous which prevents direct contact between the microbiota and IECs. Mucous is formed by different glycoproteins, the majority of which is Mucin 2 (MUC 2). Paneth cells and enterocytes secrete antimicrobial peptides (AMPs). AMPs are cationic peptides that can kill a wide range of gram-positive and gram-negative bacteria and their secretion is induced by lipopolysaccharide (LPS) and oligonucleotides. “M” cells are specialized epithelial cells of Peyer’s patches that transcytose the gut luminal antigens. Enteroendocrine cells produce the hormone serotonin which regulates intestinal inflammation and maintains immune homeostasis (Muniz et al., 2012; Yu, 2012). Apart from specialized IECs, secretory IgA (sIgA) and tight junctions (TJs) also regulate the intestinal barrier. sIgA antibody is released onto the luminal surface where it coats the inflammatory commensals and leads to their neutralization. TJs are protein complexes present on the apical surface of enterocytes. TJs limit the passage of molecules through the IECs. Disruptions in the functioning of these specialized cells and TJs result in the leaky gut that is observed in various diseases such as IBD and IBS (Lee et al., 2018). Impaired gut integrity is also reported in various bone pathologies. Li et al. (2016) showed that the gut barrier is impaired during post-menopausal osteoporosis. They reported that sex steroid deficiency modifies the expression of TJ proteins causing enhanced intestinal permeability. Increased gut permeability further leads to expansion of Th17 cells and release of osteoclastogenic cytokines such as IL-17, RANKL, and TNF-α in the intestine and bone marrow resulting in bone resorption. Nakajima et al. observed impaired gut barrier function in periodontitis (Nakajima et al., 2015). Increased intestinal barrier dysfunction was reported to be associated with the pathology of RA (Matei et al., 2021). As gut integrity regulates bone health, proper maintenance of barrier function is required for inhibition of intestinal inflammation and thus inflammatory bone loss.

**FIGURE 3:**
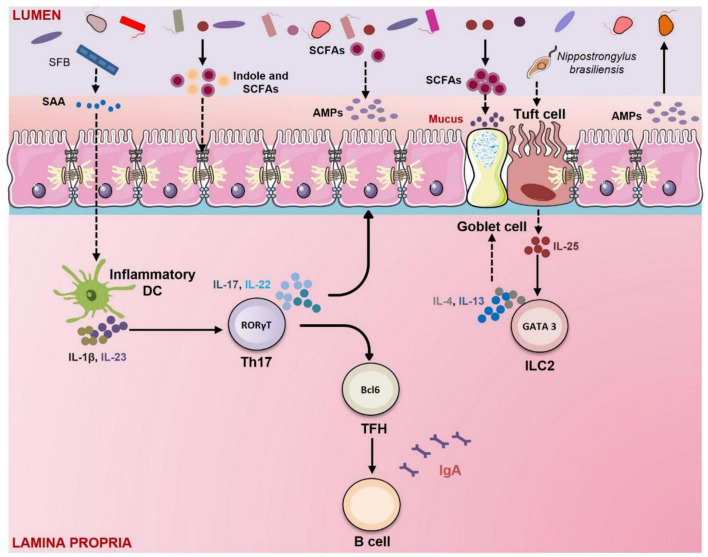
Gut membrane integrity: Role of Gut Microbiota (GM) and immune system. GM has a crucial role in regulating gut permeability. GM maintains intestinal integrity *via* several mechanisms. In mice segmented filamentous bacteria (SFB) induce the secretion of serum amyloid A (SAA) from epithelial cells which further activates the dendritic cells to secrete IL-1β and IL-23. IL-1β and IL-23 promote the differentiation of Th17 cells which produce IL-17 and IL-22. These cytokines stimulate the epithelial cells to secrete antimicrobial peptides (AMPs). GM produces tryptophan metabolites like indole that enhance the expression of tight junction (TJ) proteins. Short-chain fatty acids (SCFAs) produced by GM promote the goblet cells to produce mucus. SCFAs such as butyrate also upregulate the expression of tight junction (TJ) proteins and induce the production of AMPs from epithelial cells. Parasites like *Nippostrongylus brasiliensis* promote the secretion of IL-25 from tuft cells. IL-25 stimulates the innate lymphoid cells (ILC)-2 to produce IL-4 and IL-13. These cytokines induce goblet cell hyperplasia and mucous secretion.

Gut microbiota maintains epithelial barrier and regulates gut integrity through various mechanisms (Figure 3). GM induces mucous secretion. GF animals have less mucous secretion because of fewer and smaller goblet cells in them than the CONV-R mice (Deplancke and Gaskins, 2001). GM promotes mucous secretion through the production of SCFAs. Butyrate which is one of the SCFAs produced by microbiota promotes the expression of MUC-2 in goblet cells and induces the production of AMPs from IECs (Liu et al., 2021). GM also stimulates the expression of junctional proteins. Administration of indole that is produced by commensal microbiota from tryptophan metabolism in GF mice upregulates the expression of TJ proteins in colonic epithelial cells by activating the pregnane X receptor (Shimada et al., 2013; Venkatesh et al., 2014). SCFAs such as butyrate also upregulates the expression of TJ proteins like claudin-1 and zonula occludens-1 (Liu et al., 2021). GM is required for IgA production. GF animals have a smaller number of IgA-producing B cells (Smith et al., 2007). GM induces the secretion of AMPs. IECs release AMPs by sensing bacteria which depends on the NOD2 and myeloid differentiation primary response 88 (MyD88) signaling. NOD2 recognizes the bacterial muramyl dipeptide and provides protective immunity. Sensing of bacteria by NOD2 results in the production of AMPs such as cryptdins. NOD2 deficient mice have decreased production of AMPs and are susceptible to infection from *Listeria monocytogenes* and *Mycobacterium paratuberculosis* (Ayabe et al., 2000; Kobayashi, 2005). MyD88 is a canonical downstream adapter protein of toll-like receptor (TLR) and IL-1 receptor families. MyD88 signaling maintains mucosal homeostasis and protects against bacterial pathogens like *Citrobacter rodentium* and *Salmonella enterica* (Bhinder et al., 2014). MyD88 signaling also induces the production of AMPs by IECs. Mice lacking MyD88 in IECs have reduced production of AMPs, e.g., RegIIIγ and Defa-rs1 and have decreased mucous secretion (Bhinder et al., 2014). GM also maintains barrier integrity by inducing immune cells to produce cytokines such as IL-22. Immune cells such as Th17 cells regulate the epithelial barrier functions by secreting IL-17 and IL-22. Differentiation of Th17 cells is mainly induced by single filamentous bacteria (SFB) which adhere to the epithelial cells of the small intestine and induce the expression of serum amyloids (SAA). SAA stimulates CD11c^+^ myeloid cells to secrete IL-1β which along with other factors like IL-6 and TGF-β induce the differentiation of Th17 cells (Atarashi et al., 2011). Th17 cells then secrete the cytokines IL-22, IL-17A and IL-17F. These cytokines enhance the expression of AMPs such as β-defensin 2, S100A9, S100A7, and S100A8 (Liang et al., 2006). Parasitic helminths like *Nippostrongylus brasiliensis* stimulate IL-25 secretion from chemosensory tuft cells which are present in the gut epithelium. IL-25 activates the innate lymphoid cells (ILC)-2 to secrete IL-4 and IL-13 which further induces goblet cell hyperplasia and mucous secretion (Gerbe et al., 2016). Altogether these studies suggest that GM has a very significant role in the regulation and maintenance of gut epithelial cell integrity, which further has a role in the regulation of bone health.

### The Gut-Immune-Bone Axis

From recent studies, it is observed that there is dynamic cross-communication between the skeleton and immune system. Both the systems share the common developmental niche i.e., bone marrow. Growing awareness of the close interrelationship between the bone and immune system has stimulated the development of a new field of immunology termed “Osteoimmunology” (Walsh et al., 2006). Osteoimmunology deals with the interaction between both these systems (Dar et al., 2018a). Immune cells by secreting various cytokines influence the development of bone. Immune cells such as Tregs, Bregs, and Th17 cells have the most important role in bone regulation. Th17 cells promote bone loss by stimulating osteoclastogenesis. Th17 cells induce bone resorption directly by secreting RANKL or indirectly *via* the production of IL-17. IL-17 is an osteoclastogenic cytokine that induces expression of RANKL on osteoclastogenesis supporting cells including stromal cells and osteoblasts. Moreover, IL-17 also promotes the production of inflammatory cytokines like TNF-α, IL-1, and IL-6 which further stimulate the expression and activity of RANKL (Wang et al., 2013). On the contrary, Tregs prevent bone loss by inhibiting osteoclastogenesis. It is reported that Tregs suppress MCSF and RANKL induced osteoclastogenesis in a dose-dependent manner. Zaiss et al. showed that Tregs suppressed osteoclasts differentiation partly *via* secretion of TGF-β, IL-4, and IL-10 but majorly through a cell-cell contact-dependent manner *via* cytotoxic T lymphocyte-associated antigen 4 (CTLA-4) (Zaiss et al., 2007). On the other hand, Kim et al. showed that Tregs suppressed osteoclastogenesis in a cytokine-dependent manner and not in a cell-cell contact manner (Kim et al., 2007). Similarly, Luo et al. (2011) showed that human peripheral blood mononuclear cells (PBMCs) derived Tregs inhibited differentiation of osteoclasts *via* secretion of anti-osteoclastogenic cytokines like TGF-β and IL-10. Inflammation due to alteration in the Tregs-Th17 cell axis is one of the major factors involved in the pathogenesis of RA and periodontitis (Alunno et al., 2015; Gao et al., 2017). Studies from our lab have established the important role of the Treg-Th17 cell axis in ovariectomized (ovx) mice model (mice model for osteoporosis) (Dar et al., 2018b,c; Sapra et al., 2021c). Recently our group for the first time has established the significant role of regulatory B cells “Bregs” in inhibiting osteoclastogenesis in a contact-independent manner by secreting IL-10 thereby preventing bone loss in ovx mice model (Sapra et al., 2021b). Other immune cells such as Th1 and Th2 also regulate bone health in interferon (IFN)-γ and IL-4 dependent manner, respectively (Dar et al., 2018a). These studies point to the important role of the immune system in osteoporosis, a field with unexplored domains. To explore this interesting role of the immune system in osteoporosis, we proposed a novel field termed “Immunoporosis” which specifically deals with the role of various immune cells in the pathophysiology of osteoporosis (Srivastava et al., 2018; Sapra et al., 2021a).

**FIGURE 4:**
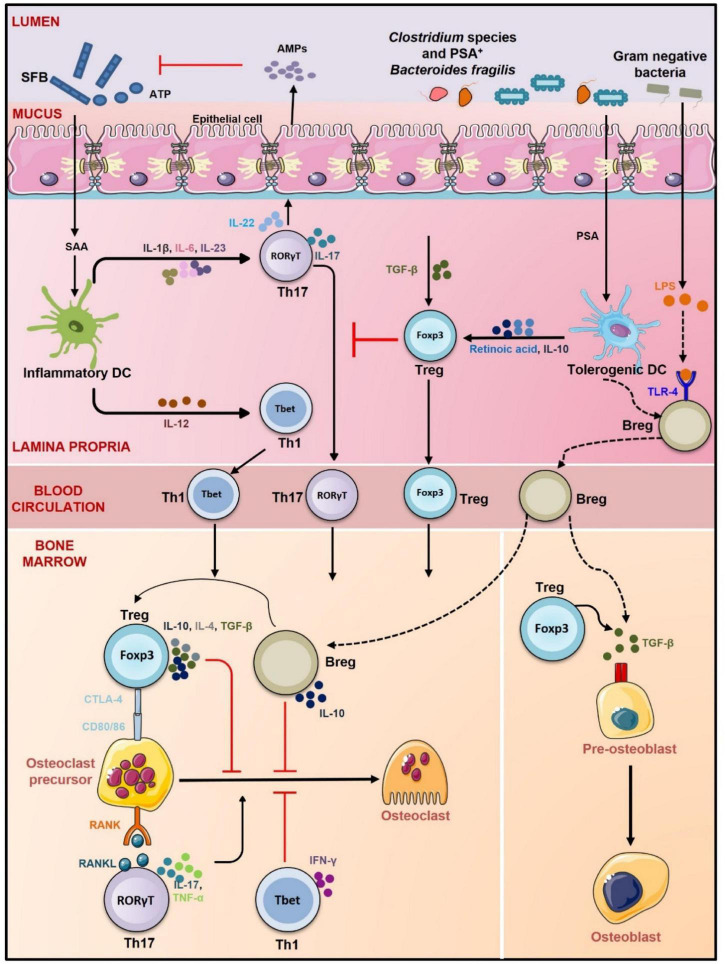
Gut-Immune-Bone axis. GM promotes the development of Treg, Th1 cells, Breg, and Th17 cells. *Clostridium* species, *Bacteroidetes fragilis*, and gram-negative bacteria promote differentiation of Tregs and Bregs *via* inducing tolerogenic DCs whereas segmented filamentous bacteria (SFB) through induction of inflammatory DCs stimulate differentiation of Th17 cells (role of SFB in inducing Th17 cells is observed in mice only). Tregs, Bregs, and Th1 cells prevent osteoclastogenesis *via* CTLA-4, IL-10, IL-4, TGF-β, and IFN-γ, respectively. Th17 cells on the other hand promote osteoclastogenesis by secreting IL-17, RANKL, and TNF-α. Tregs also induce differentiation of osteoblasts *via* TGF-β. Bregs also secrete TGF-β and thus like Tregs might also regulate osteoblastogenesis. By promoting the development of both anti-osteoclastogenic and osteoclastogenic cells, GM regulates the balance between bone formation and resorption and thus maintains bone homeostasis lines represent proposed mechanism of action.

It is reported that GM is required for the development of the immune system. Several implications in the immune system have been reported in the absence of GM in GF animals. GF animals have a reduced number of IgA secreting B cells and CD4^+^ T cells in the lamina propria. They have smaller Peyer’s patches with a reduced number of lymphoid follicles. GF animals have less developed B and T cell zones of the spleen and reduced secretion of IgG antibodies (Smith et al., 2007). On the contrary transfer of microbiota in GF animals restore the development of the immune system. It is observed that GM can maintain bone homeostasis by regulating the immune system. Several studies have shown the role of GM in the development of immune cells. It is observed that colonization of GF mice with SFB and *Clostridia*-related species induces the development of T helper cells (Gaboriau-Routhiau et al., 2009). SFB induces the differentiation of Th17 cells (Talham et al., 1999; Wu et al., 2010). SFB is reported in mice but not in humans thus its role in inducing Th17 cells is reported in mice only. However, some studies have shown the presence of SFB in humans also but its role in the induction of Th17 cells is still not clear (Chen et al., 2018; Jonsson et al., 2020). But in humans another symbiont bacteria *Bifidobacterium adolescentis* is reported that just like SFB is sufficient to induce Th17 cells (Tan et al., 2016). *Clostridium* species particularly that from the cluster IV and XIVa stimulate the differentiation of Tregs (Atarashi et al., 2011). It is reported that in healthy individuals bacterial antigens are required for expansion and generation of Tregs (Strauch, 2005) along with maintaining the balance of Tregs and Th17 cells (Ivanov and Littman, 2010). Polysaccharide A (PSA) produced by *Bacteroides fragilis* induces the differentiation of Tregs and subsequently signals the differentiated Tregs *via* TLR 2 to suppress Th17 response (Wu and Wu, 2012). GM is also required for the development of Bregs. The LPS produced by gram-negative bacteria or microbiota induced production of IL-6 and IL-1β from the dendritic cells (DCs) and macrophages induce the differentiation of Bregs (Rosser et al., 2014; Rosser and Mauri, 2015). Altogether these studies decipher the important and convincing role of GM in modulating bone health *via* regulating the host immune system i.e., the Gut-Immune-Bone axis (Figure 4).

### Gut Microbiota and Endocrine Regulation

Gut microbiota is now considered a novel endocrine organ and can maintain bone homeostasis by directing the activities of hormones which are crucial for bone regulation as discussed below:

#### Estrogen

Estrogen is the key hormone for bone development, as its deficiency results in the development of a disease condition termed post-menopausal osteoporosis. Estrogen regulates bone health either by its direct effect on the bone cells or by modulating the immune system. Estrogen suppresses bone loss by downregulating the expression of RANKL on mesenchymal cells. During estrogen deficiency, RANKL expression is enhanced on bone lining cells resulting in bone loss (Streicher et al., 2017). Luo et al. (2011) showed that estrogen prevents osteoclast differentiation by stimulating Tregs. Estrogen treatment enhances the suppressive capacity of Tregs by upregulating the expression of IL-10 and TGF-β. Estrogen also inhibits the development of Th17 cells and during estrogen deficiency differentiation of Th17 increases which promotes osteoclasts differentiation.

Gut microbiota regulates estrogen metabolism and increases the level of estrogen in the bloodstream. Estrobolome is the collection of microbial genes that are capable of metabolizing estrogen (Plottel and Blaser, 2011). Bacterial species containing the enzyme β-glucuronidases and β-glucuronides convert the conjugated form of estrogen into deconjugated form. Deconjugated estrogen enters into the circulation and binds to the estrogen receptors present on various organs such as the ovary, breast, testis, prostate, bone, and brain (Plottel and Blaser, 2011). Specific GM also processes phytoestrogens into estrogen metabolites. Phytoestrogens are polyphenols derived from the dietary compounds of plants such as soya, flaxseeds, fruits, and vegetables. Phytoestrogens have a structure similar to estrogens and thus can bind to estrogen receptors and induce estrogenic effects. Some intestinal bacteria transform phytoestrogens (e.g., isoflavones and lignans) to enterolignans which have more estrogenic activity (Bowey et al., 2003; Landete et al., 2016).

#### Parathyroid Hormone (PTH)

PTH is an 84 amino acids long polypeptide that is released from parathyroid glands. PTH regulates bone remodeling and promotes both bone formation and resorption depending on the duration of its treatment (Ma et al., 2001; Jilka et al., 2009; Silva and Bilezikian, 2015). GM has a role in the regulation of both PTH-mediated bone formation and resorption. Li et al. (2020) showed that PTH-induced bone formation is dependent on GM and PTH was unable to enhance bone mass in GF mice. Microbiota increases butyrate concentration which is required for PTH to enhance the level of Tregs in the bone marrow which then prevents bone loss. Another study by the same group reported that PTH promotes bone loss only in mice having SFB microbiota. SFB microbiota enables PTH to expand Th17 and TNF-α^+^ T cells in the gut and promote their migration from gut to bone marrow where they lead to bone loss (Yu et al., 2020).

#### Insulin-Like Growth Factor (IGF)-1

IGF-1 belongs to the family of insulin-related proteins that stimulate skeleton development. IGF-1 promotes differentiation of osteoblasts and induces bone formation in an endocrine, paracrine and autocrine fashion (Giustina et al., 2008; Locatelli and Bianchi, 2014). GM regulates the activity of IGF-1. Yan et al. showed that long-term colonization of GF mice with microbiota from CONV-R mice stimulated bone formation by increasing production of IGF-1. In contrast, antibiotic treatment of CONV-R mice suppressed bone formation and decreased the level of IGF-1 in serum. Another study reported that *L. plantarum* supplementation along with prebiotic inulin increased the expression of IGF-1 in liver (Kareem et al., 2016). *L. plantarum* was also observed to regulate bone growth in infant mice during chronic undernutrition by enhancing the synthesis and activity of IGF-1 (Schwarzer et al., 2016). In support of the above studies, Avella et al. further reported that *L. rhamnosus* administration enhanced backbone calcification in zebrafish by inducing the IGF system (Avella et al., 2012).

#### Serotonin

Serotonin is a monoamine hormone and neurotransmitter required for the regulation of various brain functions and 95% of the serotonin is synthesized in the gastrointestinal tract by the enterochromaffin cells. Serotonin is also secreted by osteoclast precursors and raphe neurons and is called bone-derived and brain-derived serotonin, respectively. Serotonin has a very significant role in the regulation of bone metabolism. Serotonin can both stimulate and inhibit bone formation (Lavoie et al., 2017). Serotonin produced by osteoclast precursors stimulates bone formation by inducing osteoblastogenesis. Brain-derived serotonin also regulates bone remodeling positively and stimulates bone formation by inhibiting the suppressive effect of sympathetic neurons on osteoblasts. However, serotonin produced in the intestine inhibits bone formation by suppressing the proliferation of osteoblasts *via* binding to the 5-HT1B receptors (Lavoie et al., 2017).

Gut microbiota upregulates serotonin synthesis in the intestine. In contrast, antibiotic treatment prevents serotonin secretion. Sjögren et al. (2012) showed that GF mice had reduced level of serum serotonin than SPF mice. Yano et al. (2015) demonstrated that colonization of GF mice with spore-forming bacteria restored the level of serum and colon serotonin to that of SPF mice. It is also reported that microbes like *Streptococcus*, *Corynebacterium*, and *E. coli* induced the synthesis of serotonin in cultures (Li L. et al., 2019). Reigstad et al. demonstrated that GM regulates serotonin synthesis by producing SCFAs. SCFAs enhance the expression of tryptophan hydroxylase (TPH1), the enzyme that produces serotonin in enterochromaffin cells (Reigstad et al., 2015).

#### Leptin

Gut microbiota regulates the gut-brain axis in part *via* the synthesis of leptin. Leptin is secreted by adipocytes and enterocytes and regulates various functions like neuroendocrine regulation, bone metabolism, and energy homeostasis (Upadhyay et al., 2015). GM enhances serum leptin levels. Queipo-Ortuño et al. (2013) observed that serum leptin level was positively correlated with the abundance of *Bifidobacterium* and *Lactobacillus* species. Another study reported that treatment of rats with antibiotic vancomycin reduced leptin in serum (Lam et al., 2012). Leptin inhibits the synthesis of brain-derived serotonin. Brain-derived serotonin binds to Ht2rC receptors present on ventromedial hypothalamic neurons and induces bone growth (Yadav et al., 2009). Leptin receptors (ObRb) are present on the raphe neurons or brainstem neurons that synthesize serotonin in the brain (Scott et al., 2009). Leptin binds to these receptors and inhibits the synthesis and release of serotonin from brainstem neurons (Yadav et al., 2011). Thus, GM-induced leptin can indirectly stimulate bone resorption by reversing the effect of brain-derived serotonin on bone growth.

### Role of Gut Associated Metabolites (GAMs) in the Regulation of Bone Health

**FIGURE 5:**
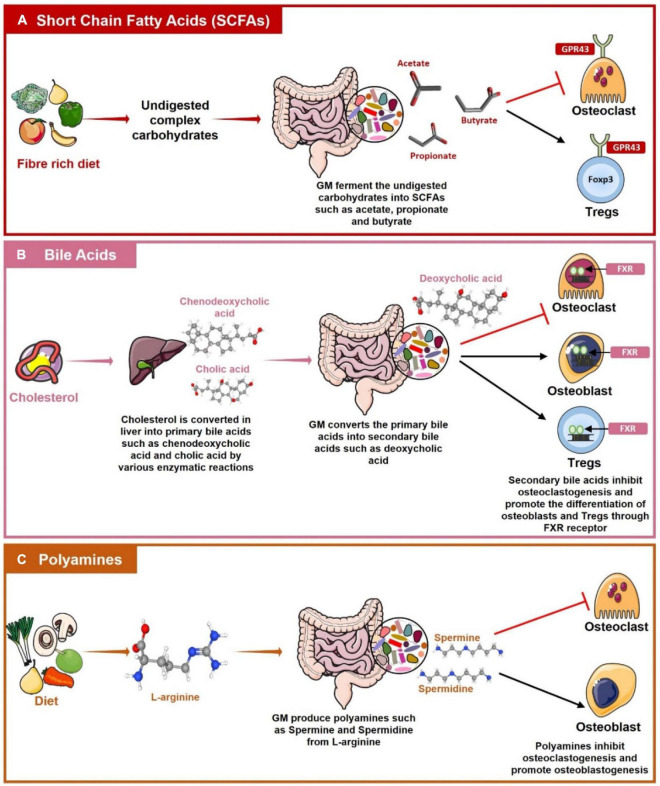
Role of Gut Associated Metabolites (GAMs) in regulating Bone Health. **(A)** Gut microbiota (GM) produces short-chain fatty acids (SCFAs) such as acetate, propionate, and butyrate by the fermentation of dietary fibers. SCFAs then regulate bone health by inhibiting osteoclastogenesis and by promoting the differentiation of Tregs with the help of G-protein coupled receptor (GPR)-43. **(B)** Primary bile acids such as cholic acid and chenodeoxycholic acid are produced in the liver from cholesterol. These primary bile acids reach the intestine where GM converts them into secondary bile acids such as deoxycholic acids. Bile acids inhibit osteoclastogenesis whereas inducing osteoblasts and Tregs differentiation by interacting with Farnesoid X receptor (FXR). **(C)** L-arginine produced from the diet is metabolized by the GM into polyamines such as Spermine and Spermidine which have a role in suppressing osteoclastogenesis and promoting osteoblastogenesis.

Metabolites produced by GM maintain bone homeostasis as discussed below (Figure 5).

#### Short-Chain Fatty Acids

**FIGURE 6:**
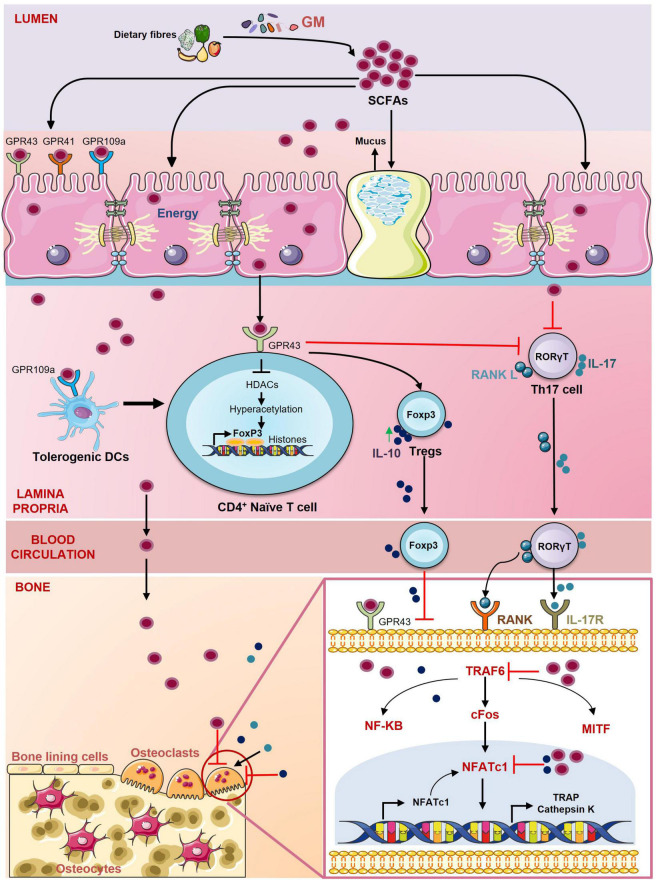
Role of short-chain fatty acids (SCFAs) in regulating Bone Health. SCFAs are the energy source for the epithelial cells and regulate the activity of various hematopoietic and non-hematopoietic cells through the G protein-coupled receptors (GPCRs) such as GPR41, GPR43, GPR109a. SCFAs promote the differentiation of Tregs through inhibition of histone deacetylases (HDACs) and suppress the secretion of IL-17 from Th17 cells. SCFAs also stimulate the development of Tregs by inducing tolerogenic DCs. SCFAs inhibit the differentiation of osteoclasts by downregulating the expression of key osteoclastogenic markers TRAF6 and NFATc1.

SCFAs are secondary metabolites produced in the colon from bacterial fermentation of undigested complex carbohydrates (Rooks and Garrett, 2016). SCFAs are of many types e.g., acetate, propionate, butyrate, pentanoate, and hexanoate. SCFAs act as an energy source not only for the GM but also for the IECs. SCFAs regulate a diverse range of physiological functions related to human health. SCFAs are ligands for G protein-coupled receptors (GPCRs) and regulate the activity of various hematopoietic and non-hematopoietic cells through GPCRs. Regulatory activity of SCFAs requires signaling through GPCRs primarily *via* GPR43 (also named as FFAR2), GPR41 (also named as FFAR3), and GPR109A (also named as HCAR2) (Sivaprakasam et al., 2016). SCFAs are controllers of osteoclast metabolism. It is reported that supplementation of either a high fiber diet or SCFAs to mice prevented postmenopausal bone loss and RA by inhibiting osteoclasts differentiation. SCFAs (propionate and butyrate) treatment downregulate the expression of osteoclast genes such as tumor necrosis factor receptor-associated factor 6 (TRAF6) and nuclear factor of activated T-cells (NFATc1) resulting in decreased osteoclastogenesis (Lucas et al., 2018). SCFAs prevent bone loss by interacting with the FFAR2 receptor present on osteoclasts. Deterioration of maxillary bone was observed in FFAR2 deficient mice. Administration of a high fiber diet on the other hand partially restored the bone damage. *In vitro* increased osteoclastogenesis was observed in bone marrow cells derived from FFAR2 deficient mice whereas treatment with SCFAs and FFAR2 agonist phenylacetamide-1 prevented osteoclastogenesis (Montalvany-Antonucci et al., 2019). SCFAs regulate fibroblast activity and it is observed that propionate supplementation has a role in mitigating arthritis by preventing synovial fibroblasts mediated production of inflammatory mediators (Friščić et al., 2021). Apart from regulating the activity of osteoclasts, SCFAs also maintain immune homeostasis (Figure 6). SCFAs promote the induction of Tregs and inhibit the development of Th17 cells (Asarat et al., 2016). SCFAs induce differentiation of Tregs by interacting with the GPR43 receptor present on CD4^+^ T cells (Smith et al., 2013; Rooks and Garrett, 2016). SCFAs are also inhibitors of histone deacetylases (HDACs). It is observed that treatment of naïve T cells with butyrate under Tregs polarizing conditions increased histone H3 acetylation in the promoter and conserved non-coding sequence of Foxp3 gene resulting in differentiation of Treg (Furusawa et al., 2013). Another SCFA acetate promoted acetylation of Foxp3 through inhibition of HDAC9 (Thorburn et al., 2015). SCFAs induced Tregs thereby alleviate bone loss by suppressing osteoclasts differentiation. These Tregs also inhibit bone loss by interacting with CD8^+^ T cells. Tyagi et al. reported that butyrate-induced Tregs stimulate CD8^+^ T cells in the bone marrow to secrete Wnt ligand Wnt10b. Wnt10b then further induces bone formation by activating the Wnt signaling in osteoblasts (Tyagi et al., 2018). Butyrate administration suppressed arthritis in mice by inducing Bregs. Butyrate supplementation enhances the production of 5-Hydroxyindole-3-acetic acid (HIAA), an AhR ligand that programs B cells to become Bregs which mitigate inflammatory arthritis (Rosser et al., 2020). Also, SCFAs can positively influence bone healing process by regulating the activity of bone cells and thus can be employed for fracture healing (Wallimann et al., 2021). Collectively, all these studies clearly establish that SCFAs supplementation can be exploited as a potential therapeutics for the management of various bone pathologies including osteoporosis, fractures and RA.

#### Bile Acids

Bile acids are surfactants or emulsifying agents that enhance the absorption and digestion of lipids. Primary bile acids such as cholic acid and chenodeoxycholic acid are produced in the liver by hepatocytes from cholesterol by several enzymatic reactions. Primary bile acids are then further processed by GM into secondary bile acids like deoxycholic and lithocholic acid in the colon where they regulate various metabolic pathways (Staels and Fonseca, 2009). Several studies have shown the role of bile acids in the regulation of bone health. Decreased expression of G-protein coupled bile acid receptor 5 (Tgr5) is reported on PBMCs of RA patients as compared to healthy controls. Expression of Tgr5 was found to be negatively correlated with disease severity. On the other hand, treatment with lithocholic acid has been reported to decrease arthritis score in collagen II-induced arthritis mouse model along with suppressing inflammatory responses in RA patients (Li Z. et al., 2019). Zhao et al. reported that bile acids are positively correlated with BMD as osteoporotic and osteopenic post-menopausal women have significantly reduced levels of bile acids than healthy controls (Zhao et al., 2020). It is observed that bile acids induce differentiation of osteoblasts *via* stimulation of farnesoid X receptor (FXR). Activation of FXR upregulates Runx2 and β catenin expression and thus promotes osteoblastogenesis (Cho et al., 2013). Bile acids also inhibit osteoclastogenesis by downregulating c-Fos and NFATc1 expression *via* FXR activation (Zheng et al., 2017). Li et al. showed that bile acids can inhibit osteoclastogenesis *via* induction of Tgr5. Tgr5 stimulation prevents osteoclast differentiation by increasing phosphorylation of AMP-activated kinase (AMPK) (Li S.-C. et al., 2019). They also reported that activation of both Tgr5 and FXR can prevent estrogen-induced bone loss (Li Z. et al., 2018). Bile acids are important modulators of the host immune system. It is observed that bile acids induce differentiation of Tregs by enhancing Foxp3 expression and suppressing differentiation of Th17 cells by directly binding to RORγt (Hang et al., 2019). Thus, bile acids also have the potential in regulating bone health *via* modulating the host immune system.

#### Polyamines

Polyamines are polycations that are required for various biological processes in the body such as cell growth, proliferation, and survival (Minois et al., 2011). The three main polyamines are spermine, spermidine and putrescine. Polyamines are required for the proper maintenance of bone health. It is reported that polyamine (spermidine and spermine) supplementation inhibits ovx induced bone loss *via* suppression of osteoclasts differentiation and proliferation (Yamamoto et al., 2012). Yamada et al. reported that daily supplementation of polyamines rich *Saccharomyces cerevisiae* S631 significantly prevented osteoclasts activation and bone loss in ovx mice (Yamada et al., 2019). Daily administration of spermine to rats ameliorated bone and cartilage destruction due to collagen-induced arthritis (Iezaki et al., 2012). Albert et al. (2015) reported that specific alterations in polyamines metabolism led to Snyder-Robinson Syndrome (SRS), a disease characterized by profound depletion of osteoclasts and osteoblasts. Recently, it has also been reported that warmth exposure prevents osteoporosis in mice by inducing the production of polyamines. Polyamines inhibit the differentiation of osteoclasts whereas promoting the differentiation of osteoblasts (Chevalier et al., 2020). Polyamines like spermidine modulate the immune system and have a role in the prevention of arthritis *via* inhibiting the polarization of inflammatory M1 macrophages (Yuan et al., 2021). Polyamines also have a very important role in maintaining gut integrity. They regulate the epithelial barrier of the gut by activating the transcription factor c-Myc which then upregulates the expression of TJ protein E-cadherin (Liu et al., 2009). Polyamines also regulate the gut barrier by stimulating the expression of TLR2 on IECs. It is reported that in the absence of polyamines expression of TLR2 decreases on IECs resulting in enhanced gut permeability (Chen et al., 2007).

Based on the above studies, it can be concluded that GM has a crucial role in the development of bone health and thus dysregulation of GM can lead to various inflammatory bone pathologies. Various drugs are currently being employed for the treatment of different bone disorders but restoring the dysregulated GM would be a path-breaking approach in managing various bone pathologies. GM can be modulated by diet, antibiotics, and drugs. Dietary interventions are the current favorite methods for the manipulation of GM. Dietary interventions include probiotics, prebiotics, synbiotics, and postbiotics. Biotics is the most favorable therapy for GM-associated disorders and can be easily adopted for preventing various bone pathologies. In the following sections, we will now review the possibility of employing dietary inventions as novel therapeutics for the treatment and management of various inflammatory bone pathologies.

## Dietary Interventions and Modulation of Gut Microbiota

Dietary interventions that are most commonly in use for modulation of GM are discussed below:

### Probiotics

Probiotics are live microorganisms which when administered in adequate amounts confer health benefits on the host. Probiotics are contained in various dietary supplements and fermented foods like yogurt, cheese, kefir, wine, bread, and kumis (Ozen and Dinleyici, 2015). The most widely used probiotics are *Lactobacillus* and *Bifidobacterium* species (Gupta and Garg, 2009). Probiotics are found effective against various clinical manifestations such as IBD, IBS, diabetes, obesity, and non-alcoholic fatty liver disease (Gupta and Garg, 2009; Verna and Lucak, 2010; Plaza-Diaz et al., 2019). Recently various studies have shown the role of probiotics in preventing bone disorders. One of the mechanisms *via* which probiotics attenuate bone loss is by preventing alterations in the GM composition. Dysbiosis is reported in various bone diseases. It is reported that the abundance of *Firmicutes* is significantly higher whereas that of *Bacteroidetes* is significantly lower in osteoporotic patients (Wang et al., 2017). Another study reported that osteoporotic patients have significantly higher proportions of *Faecalibacterium* and *Dialister* genera (Xu et al., 2020). Altered *Firmicutes*/*Bacteroidetes* ratio is also observed in RA, osteoarthritis, and periodontitis (Lourenςo et al., 2018; Lee et al., 2019). Probiotics supplementation prevents the growth of various pathogenic bacteria thereby restoring the composition of gut flora and thus preventing dysbiosis-mediated bone loss. Studies have shown the role of probiotics like *Bifidobacterium* spp. and *Lactobacillus reuteri* in preventing dysbiosis-associated bone loss (Achi et al., 2019; Schepper et al., 2019). Probiotics also inhibit bone loss by preventing inflammation. Several studies including our own have shown the role of inflammation in bone loss. It is observed that the Tregs-Th17 cell axis alters during various bone diseases like osteoporosis, RA, osteoarthritis and periodontitis (Leipe et al., 2010; Kikodze et al., 2016; Gao et al., 2017; Li et al., 2017). Studies from our lab and other have shown that supplementation of probiotics such as *Lactobacillus acidophilus*, *Bacillus clausii*, and *Lactobacillus rhamnosus* prevent bone loss by restoring the pivotal “Treg-Th17 cell” balance, an important determinant of bone loss in osteoporosis (Dar et al., 2018b,c; Sapra et al., 2021c). Probiotics have also been reported in directly regulating bone cells e.g., *Lactobacillus reuteri* 6475 and *Lactobacillus rhamnosus* have a role in stimulating osteoblastogenesis along with inhibiting the development of osteoclasts (Quach et al., 2019; Sapra et al., 2021c). Probiotics such as *Bifidobacterium longum* and *Lactobacillus reuteri* 6475 enhance bone formation by decreasing gut permeability and increasing the absorption of Ca and vitamin D (Rodrigues et al., 2012; Röth et al., 2019).

### Prebiotics

Prebiotics are defined as “non-digestible food ingredient that beneficially affects the host by selectively stimulating the growth and activity of one or a limited number of bacteria in the colon, and thus improves host health” (Gibson et al., 2010). Common prebiotics is galactooligosaccharides (GOS), fructooligosaccharide (FOS), and trans-galactooligosaccharide (TOS). Prebiotics act as a nutrient source for GM and their degradation results in the formation of SCFAs (Davani-Davari et al., 2019). Prebiotics can selectively influence and modify GM composition by promoting the growth of certain microorganisms. Several recent studies have shown that prebiotics such as Konjoc oligosaccharides, GOS, FOS can significantly enhance bone mass by reversing dysbiosis (Ai et al., 2021; Zhang et al., 2021). Prebiotics also enhance bone mass by decreasing intestinal permeability and thus preventing systemic inflammation (Zhang et al., 2021). Xylo-oligosaccharides (XOS) treatment was observed to increase BMD by upregulating the expression of Ca transporters in the intestine (Gao and Zhou, 2020). It is reported that FOS also enhances osteogenesis and thus induces bone formation.

### Synbiotics

Synbiotics are a mixture of probiotics and prebiotics. In May 2019, the International Scientific Association for Probiotics and Prebiotics defined synbiotics as “a mixture compromising live microorganisms and substrates selectively utilized by host microorganisms that confers a health benefit on the host”. The concept of synbiotics has emerged nearly 25 years ago where it is observed that probiotics and prebiotics can be combined. Synbiotics increase the survival and implantation of live microbial dietary supplements. Synbiotics are divided into complementary and synergistic synbiotics. In Complementary synbiotics both the probiotics and prebiotics work independently to provide health benefits to the host. However, in the synergistic approach, the substrate or the prebiotic enhances the functionality of the probiotics and both prebiotics and probiotics work synergistically to bring health benefits (Swanson et al., 2020). Common probiotics such as *Lactobacillus*, *Bifidobacterium*, and *Bacillus* species and prebiotics like FOS, XOS, and GOS are used for making symbiotic formulations (Pandey et al., 2015). Synbiotics enhance Ca absorption by increasing the expression of Ca-binding proteins. In postmenopausal women, it is observed that administration of fermented plasma milk enhanced oral bioavailability and concentration of isoflavones in plasma. Isoflavones are known to decrease the risk of osteoporosis (Ilesanmi-Oyelere and Kruger, 2020). Synbiotics treatment prevented bone loss in the high fat diet-fed rats by preventing osteoclastogenesis and increasing bone formation activities (Eaimworawuthikul et al., 2020). It is observed that synbiotics administration enhanced the level of bone mineral content, BMD, and bone area in broilers subjected to cyclic heat stress episodes (Yan et al., 2019). In rats, it is reported that administration of Yacon flour with *B. longum* increased the mineral contents of bone (Rodrigues et al., 2012).

### Postbiotics

**FIGURE 7:**
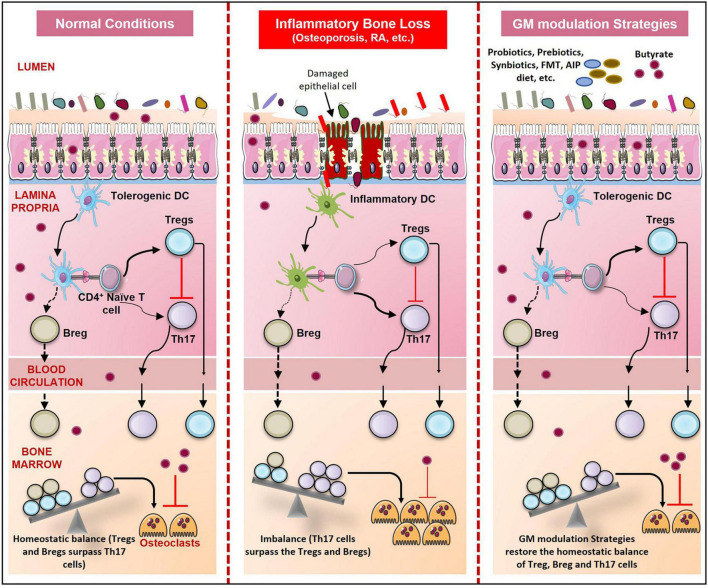
GM modulation Strategies and Skeletal Health. During normal conditions, immune homeostasis is maintained. But in bone pathologies like osteoporosis, RA, and periodontitis due to dysbiosis, gut permeability increases resulting in an increase in the number of inflammatory Th17 cells. These Th17 cells outcompete the regulatory Tregs and Bregs which result in enhanced osteoclastogenesis and thus bone loss. Probiotics, prebiotics, synbiotics, postbiotics, FMT, Orthogonal niche engineering, autoimmune protocol diet (AIP), and Personalized GM modulation strategies prevent dysbiosis and restore the homeostatic balance of immune and bone cells.

Postbiotics is a relatively new term introduced in the – ‘biotics’ field. Postbiotics are functional bioactive compounds comprising of soluble factors such as metabolites and cell wall components released by the probiotics. Postbiotics constitute metabolic by-products such as SCFAs or factors released upon bacterial lysis such as teichoic acid, polysaccharides, peptidoglycan derived muropeptides, enzymes, peptides, cell surface proteins, and organic acids (Aguilar-Toalá et al., 2018). Jung et al. and Sapra et al. reported that culture supernatants of probiotics prevented RANKL induced osteoclastogenesis. Postbiotics also have a role in the prevention of bone loss. Recently, it is reported that supplementation of ovariectomized rats with lysates and supernatant of *L. casie*, *B. longum*, *B. cougulans*, *L. acidophilus*, and *L. reuteri* significantly increased BMD (Montazeri-Najafabady et al., 2019). Culture supernatants of probiotics prevented osteoclastogenesis of RAW 264.7 macrophages (Jung et al., 2021). We also reported that the culture supernatant of *L. rhamnosus* suppressed osteoclastogenesis *in vitro* (Sapra et al., 2021c). Collins et al. reported that secreted factors of *L. reuteri* prevented bone loss by modulating the immune system (Collins et al., 2019). As discussed earlier SCFAs which are secondary metabolites produced by GM prevent bone loss by modulating the immune system and inhibiting osteoclastogenesis (Lucas et al., 2018; Tyagi et al., 2018). Altogether, probiotics, prebiotics, synbiotics, and postbiotics are promising candidates for being employed as therapeutics in inhibiting inflammatory bone loss *via* various mechanisms (Figure 7 and Table 1).

**TABLE 1:** Role of various Biotics in regulating bone health.

S. No.	Name	Animal species used	Findings	Disease	References
1.	*L. helveticus*	Sprague-Dawley rats	↑BMD ↑Bone strength	Osteoporosis	Narva et al., 2007
2.	*L. casei*	Wister rats	↓Arthritis score ↓Knee inflammation	Arthritis	Amdekar et al., 2012
3.	Yacon Flour and *Bifidobacterium longum*	Wister rats	↑Ca ↑P ↑Mg	-	Rodrigues et al., 2012
4.	*L. reuteri* ATCC PTA 6475	Balb/c mice	↑BV/TV ↑Tb. N ↑Tb. Th ↓Osteoclast activity	Osteoporosis	Britton et al., 2014
5.	*Bifidobacterium longum*	Sprague-Dawley rats	↑Serum osteocalcin ↑BV/TV ↑Tb. N ↑Tb. Th ↑BMD ↑Strength of femur	Osteoporosis	Parvaneh et al., 2015
6.	*L. rhamnosus* GG and VSL#3	C57BL6/J mice	↑BV/TV ↑Tb. N ↑TB. Th ↑Osteocalcin ↓Tb. Sp	Sex steroid deficiency	Li et al., 2016
7.	*L. casei* Shirota	Elderly patients	↓DASH score ↓Pain	Fracture	Lei et al., 2016
8.	Red clover extract rich in isoflavone aglycones and probiotics	Human females	↑BMD	Post-menopausal osteoporosis	Lambert et al., 2017
9.	*L. casei* Shirota	Human male and females	↓Serum hs-CRP	Knee osteoarthritis	Lei et al., 2017
10.	*Bacillus clausii*	BALB/c mice	↑BV/TV ↑Tb. Th ↑Tb. N ↓ Tb. S	Post-menopausal osteoporosis	Dar et al., 2018b
11.	*L. acidophilus*	BALB/c mice	↑BV/TV ↑Tb. Th ↑Tb. N ↓Tb. S	Post- menopausal osteoporosis	Dar et al., 2018c
12.	SCFAs	C57BL6/J mice	↑Osteoclastogenesis	Post-menopausal osteoporosis	Lucas et al., 2018
13.	*Bacillus subtilis* C-3102	Post-menopausal women	↑BMD	Post-menopausal osteoporosis	Takimoto et al., 2018
14.	*L. reuteri* 6475	Elderly women	↑BMD ↓TRACP-5b	Age-associated bone loss	Nilsson et al., 2018
15.	Fructooligosaccharides and mixture of *Enterococcus faecium*, *Pediococcus acidilactici*, *Bifidobacterium animalis*, and *L. reuteri*	Broilers	↑BMD ↑Bone area	Cyclic heat stress episodes	Yan et al., 2019
16.	*Lactobacillus paracasei* DSM 13434, *Lactobacillus plantarum* DSM 15312, and *Lactobacillus plantarum* DSM 15313	Post-menopausal women	↑BMD	Post-menopausal osteoporosis	Jansson et al., 2019
17.	XOS	ICR mice	↑BMD	-	Gao and Zhou, 2020
18.	*L. rhamnosus*	BALB/c mice	↑BV/TV ↑Tb. Th ↑Tb. N ↓Tb. S	Post- menopausal osteoporosis	Sapra et al., 2021c
19.	Lysates and supernatant of *B. coagulans*, *B. longum*, *L. acidophilus*, *L. rueteri*	Sprague-Dawley rats	↑BMD	Postmenopausal osteoporosis	Montazeri-Najafabady et al., 2019
20.	Culture supernatant of *L. salivarius* MG4265	-	↑Osteoclastogenesis	-	Jung et al., 2021

*BMD, Bone mineral density; BV/TV, Bone volume over total volume; Tb. Th, Trabecular thickness; Tb. N, Trabecular number; Tb. S, Trabecular spacing; Ca, Calcium; P, Phosphorus; Mg, Magnesium; SCFAs, Short-chain fatty acids; GOS, Galactooligosaccharides; FOS, Fructooligosaccharide; XOS, Xylooligosaccharides.*

### Orthogonal Niche Engineering

Apart from biotics, various other dietary approaches are used to modulate the GM composition. Recently a new technique called orthogonal niche engineering is harnessed to manipulate the GM. In orthogonal niche engineering microbes and substrate relationship is leveraged by using customized niche for an introduced bacterium. In prebiotics and synbiotics we generally use the common substrates that lead to a momentary increase in the organism’s population but not the persistent engraftment of the organisms. However, in the case of orthogonal niche engineering, uncommon substrates are used. For example, Kearney et al. used a seaweed polysaccharide porphyrin to stably engraft the human commensal bacteria *B. plebeius* DSM 17135 to the mice’s gut. The introduction of seaweed makes it exclusively accessible to the bacteria, enabling it to colonize and compete with the native commensals. Thus, orthogonal niche engineering provides a novel opportunity for the stable engraftment of microbes in the gut environment (Kearney et al., 2018; Wolter et al., 2021). As various probiotics are lost from the gut after a particular period, orthogonal niche engineering can be harnessed for stable engraftment of these probiotics (Wolter et al., 2021). But currently, studies on orthogonal niche engineering are limited and its role for the management of bone pathologies still needs to be explored.

### Autoimmune Protocol (AIP) Diet

As dietary changes result in various autoimmune and inflammatory disorders, formulation of diet that prompts the restoration of immune homeostasis, termed as autoimmune protocol (AIP) diet is also proposed. AIP diet is a version of the paleo diet designed to avoid the inclusion of food that can trigger an unnecessary immune response. As a modern diet is not able to support the healthy microbiome, it results in compromised development of immune system (Singh et al., 2017). AIP diet controls the immune response and evidence supports its role in reducing inflammation and thus can be useful in the treatment of several inflammatory disorders (Wolter et al., 2021). As dysbiosis-associated immune responses promote bone resorption, AIP can also be a potential therapy for preventing inflammatory bone loss. But as there are not many studies in support of the AIP diet, well-designed investigations and clinical trials are required to study the effect of the AIP diet in the context of bone pathologies.

### Fecal Microbiota Transplantation

Fecal microbiota transplantation (FMT) also holds the potential along with the dietary supplements in preventing GM-associated pathologies. In FMT, the fecal content of the healthy individual is transferred to the recipient having the perturbed GM resulting in the re-establishment of a healthy microbiome. FMT was first used for the treatment of *Clostridioides difficile* infection and was effective in treating 90% of the patients. After that FMT was explored for the management of several disorders like multiple sclerosis, RA, type 1 diabetes mellitus, IBD, Crohn’s disease, ulcerative colitis, liver diseases, and various other metabolic disorders. Results from these studies gave mixed responses and the effectiveness of FMT in these disorders is still controversial (Wargo, 2020; Wolter et al., 2021). Dysbiosis is associated with various bone pathologies and FMT can be exploited as a potential tool for restoration of perturbed GM in these pathologies (Xu et al., 2020). A recent study has shown the potential of FMT in preventing bone loss in a rat model of senile osteoporosis. It is reported that the transplantation of GM from healthy rats to aged rats having senile osteoporosis attenuated the bone loss by preventing dysbiosis in aged rats. After 24 weeks of FMT in aged rats, there was a significant increase in the bone parameters such as bone volume fraction, trabecular number, and thickness. FMT also increased the expression of TJ proteins like occludin and claudin in the recipient rats (Ma et al., 2021). This study shows the efficacy of FMT in preventing bone loss but still, there is controversy regarding the efficacy of FMT. Therefore, we proposed that a combination of FMT with dietary supplementations like probiotics and synbiotics can be another approach for the management of these inflammatory bone conditions. Giving a similar diet to the recipient as taken by the donor along with FMT can also be considered as a strategy to increase the efficacy of FMT.

### Personalized Gut Microbiota Modulation Strategies

Modulation of GM by the above-used techniques is currently employed to prevent the pathogenesis of several disorders but mechanisms of their working are still not completely clear. Modulation of GM is giving varied results with less reproducibility. One reason could be the specific microbiome of every individual and thus one approach used for a person may not work for another person i.e., one size does not fit all. Therefore, a personalized approach could be encouraged *via* modulation of GM according to the need of the individual taking into consideration its health status and diet regime. As dysbiosis is observed in all types of bone pathologies such as RA, osteoporosis, and periodontitis sequencing of an individual’s microbiome and then devising a GM modulation strategy according to the need of the individual with biotics would be a promising approach for management of GM associated bone pathologies.

## Conclusion

Various studies are now supporting and establishing the role of GM in maintaining bone homeostasis. GM regulates bone metabolism by various mechanisms such as inducing the development of the immune system, maintaining gut permeability, and regulating the endocrine system. Microbial metabolites can also regulate bone health by directly affecting the process of bone remodeling or modulating the host immune system. Alteration in GM composition leads to various bone pathologies such as osteoporosis, RA, and periodontitis. These skeleton manifestations are affecting the lives of millions of people. Thus, interventions targeting the GM can be a promising approach both for the prevention and management of various bone pathologies. GM can be modulated by the use of antibiotics, diet, and drugs. Recent studies have shown that biotics (probiotics, prebiotics, synbiotics, and postbiotics) can prevent various bone disorders. They restore the normal GM composition and prevent bone resorption through several established mechanisms. They primarily regulate the immune system, intestinal barrier functions, and activity of bone cells along with enhancing Ca and vitamin D absorption. Therefore, probiotics, prebiotics, synbiotics, and postbiotics can be exploited as an effective and safer therapies for the management of various bone-related ailments. However, clinical trials at a large scale are needed to further delineate their efficacy in humans. Apart from biotics, orthogonal niche engineering, AIP diet, and FMT along with personalized GM modulation strategies could also be employed as promising therapeutic modalities in the management and treatment of various inflammatory bone disorders and therefore their role in skeleton health should be explored on priority.

## Author Contributions

RS contributed to the conceptualization and writing of the manuscript. AB participated in the writing and editing of the review. LS, AT, PM, and SS provided valuable inputs in the preparation of the manuscript. AB created the illustrations. All authors contributed to the article and approved the submitted version.

## Conflict of Interest

The authors declare that the research was conducted in the absence of any commercial or financial relationships that could be construed as a potential conflict of interest.

## Publisher’s Note

All claims expressed in this article are solely those of the authors and do not necessarily represent those of their affiliated organizations, or those of the publisher, the editors and the reviewers. Any product that may be evaluated in this article, or claim that may be made by its manufacturer, is not guaranteed or endorsed by the publisher.

## Abbreviations

GM: Gut Microbiota
IEC: Intestinal Epithelial Cells
SCFAs: Short Chain Fatty Acids
AhR: Aryl hydrocarbon Receptors
IBD: Inflammatory Bowel Disease
IBS: Inflammatory Bowel Syndrome
MSC: Mesenchymal Stem Cells
Runx2: Runt Related Transcription Factor 2
PTH: Parathyroid Hormone
IGF-1: Insulin like Growth Factor-1
MCSF: Macrophage Colony Stimulating Factor
RANKL: Receptor Activator of Nuclear Factor kappa- B Ligand
TRAP: Tartrate Resistant Acid Phosphatase
TNF: Tumor Necrosis Factor
IL: Interleukin
OPG: Osteoprotegerin
Ca: Calcium
RA: Rheumatoid Arthritis
CONV-R: Conventionally Raised
NOD: Nucleotide-binding Oligomerization Domain
BMMCs: Bone Marrow derived Macrophages
SPF: Specific Pathogen Free
GH: Growth Hormone
GAMs: Gut Associated Metabolites
M cells: Microfold cells
AMP: Anti-Microbial Peptides
MUC: Mucin
LPS: Lipopolysaccharide
sIgA: secretory IgA
TJ: Tight Junctions
TGF: Transforming Growth Factor
IFN: Interferon
PSA: Polysaccharide A
TLR: Toll Like Receptor
DC: Dendritic Cells
TPH: Tryptophan Hydroxylase
HDAC: Histone Deacetylase
GPCR: G Protein Coupled Receptor
BMD: Bone Mineral Density
FXR: Farnesoid X Receptor
Tgr5: G Protein Coupled Bile Acid Receptor 5
AMPK: AMP activated Protein Kinase
FOS: Fructooligosaccharides
XOS: Xylooligosaccharides
GOS: Galactooligosaccharides
AIP: Autoimmune Protocol
FMT: Fecal Microbiota Transplantation.
